# Spike Gene Evolution and Immune Escape Mutations in Patients with Mild or Moderate Forms of COVID-19 and Treated with Monoclonal Antibodies Therapies

**DOI:** 10.3390/v14020226

**Published:** 2022-01-24

**Authors:** Aude Jary, Stéphane Marot, Antoine Faycal, Sacha Leon, Sophie Sayon, Karen Zafilaza, Emna Ghidaoui, Stéphanie Nguyen Quoc, Safaa Nemlaghi, Sylvain Choquet, Martin Dres, Valérie Pourcher, Vincent Calvez, Helga Junot, Anne-Geneviève Marcelin, Cathia Soulié

**Affiliations:** 1 Institut Pierre Louis d’Epidémiologie et de Santé Publique (iPLESP), INSERM, Sorbonne Université, 75013 Paris, France; stephanesylvain.marot@aphp.fr (S.M.); sophie.sayon@aphp.fr (S.S.); karen.zafilaza@iplesp.upmc.fr (K.Z.); emna.ghidaoui01@gmail.com (E.G.); vincent.calvez@aphp.fr (V.C.); anne-genevieve.marcelin@aphp.fr (A.-G.M.); cathia.soulie-ext@aphp.fr (C.S.); 2 AP-HP, Pitié-Salpêtrière, Service de Virologie, Bâtiment CERVI, 47-83 Boulevard de l’Hôpital, 75013 Paris, France; 3 AP-HP, Hôpital Pitié-Salpêtrière, Service de Maladie Infectieuses et Tropicales, 75013 Paris, France; antoine.faycal@aphp.fr; 4 Service de Pharmacie, AP-HP, Sorbonne Université, Hôpital Pitié-Salpêtrière, 75013 Paris, France; sacha.leon@aphp.fr (S.L.); helga.junot@aphp.fr (H.J.); 5 Service d’Hématologie Clinique, AP-HP, Sorbonne Université, Hôpital Pitié-Salpêtrière, 75013 Paris, France; stephanie.nguyen-quoc@aphp.fr (S.N.Q.); sylvain.choquet@aphp.fr (S.C.); 6 Service de Médecine Intensive–Réanimation (Département “R3S”), AP-HP. Sorbonne Université, Hôpital Pitié-Salpêtrière, 75013 Paris, France; safaa.nemlaghi@aphp.fr (S.N.); martin.dres@aphp.fr (M.D.); 7 UMRS1158 Neurophysiologie Respiratoire Expérimentale et Clinique, INSERM, Sorbonne Université, 75013 Paris, France; 8 Service de Maladie Infectieuses et Tropicales, Institut Pierre Louis d’Epidémiologie et de Santé Publique (iPLESP), INSERM, AP-HP, Sorbonne Université, Hôpital Pitié-Salpêtrière, 75013 Paris, France; valerie.martinez@aphp.fr

**Keywords:** COVID-19, SARS-CoV-2, monoclonal antibodies therapy, spike gene, immune escape mutation, Q493R

## Abstract

We explored the molecular evolution of the spike gene after the administration of anti-spike monoclonal antibodies in patients with mild or moderate forms of COVID-19. Four out of the 13 patients acquired a mutation during follow-up; two mutations (G1204E and E406G) appeared as a mixture without clinical impact, while the Q493R mutation emerged in two patients (one receiving bamlanivimab and one receiving bamlanivimab/etesevimab) with fatal outcomes. Careful virological monitoring of patients treated with mAbs should be performed, especially in immunosuppressed patients.

## 1. Background

The severe acute respiratory syndrome coronavirus 2 (SARS-CoV-2) has caused a global pandemic and continues to spread around the world. The coronavirus disease 2019 (COVID-19) can display a wide range of symptoms, from asymptomatic infection to mild and moderate illness, to acute respiratory distress syndrome and, ultimately, especially in vulnerable patients, lead to death [1]. In France, since early 2021, the vaccination campaign has been ongoing, and it was firstly offered to people with a high risk for progressing to a severe form of COVID-19, including people aged >60 years, and those with diabetes, cardiovascular or lung diseases, active cancers under chemotherapy or undergoing transplantation. However, some of them, such as immunosuppressed patients, never developed (or developed a low) immunological response, and thus remain at risk of COVID-19 [2,3].

Meanwhile, the antiviral drugs available to manage productive SARS-CoV-2 infection are limited, and their ability to treat and prevent COVID-19 progression remains under debate [4,5]. In severe cases, convalescent plasma and immunosuppressors have also been explored and results are mixed [6,7]. The neutralizing monoclonal antibodies (mAbs) were developed to target specifically the surface spike (S) glycoprotein of SARS-CoV-2 that mediates viral entry into host cells. Currently, three mAbs therapies are available, i.e., bamlanivimab alone (LY-CoV555, Eli Lilly), bamlanivimab + etesevimab (Eli Lilly) and casirivimab + imdevimab (REGN-COV2, Regeneron Pharmaceuticals and Roche Holding). Although the BLAZE-1 trial showed that both monotherapy with bamlanivimab and combination therapy with bamlanivimab and etesevimab reduced the risk of hospitalization and COVID-19 progression compared to a placebo group [8], some concerns remain over the risk of mutation emergence in the S gene under selective pressure from monotherapy with bamlanivimab [9]. Some cases of Q493R mutations following bamlanivimab/etesevimab administration were also reported in the literature and are associated with a reduced viral clearance and, in some patients, with fatal outcome [10,11,12,13].

In the present study, we aimed to describe the evolution of the spike gene sequence in patients treated with mAbs therapy to prevent progression to a severe form of COVID-19.

## 2. Methods

This monocentric study included patients with mild or moderate forms of COVID-19 who received anti-spike neutralizing mAbs to prevent the progression of the disease and who were followed at the Pitié-Salpêtrière hospital, Paris, France. Patients were eligible for mAbs treatment if they met the criteria of the French Ministry of Health (https://solidarites-sante.gouv.fr/soins-et-maladies/maladies/maladies-infectieuses/coronavirus/tout-savoir-sur-la-covid-19/article/traitement-par-anticorps-monoclonaux; accessed on 24 February 2021), and those who received mAbs from March to June 2021 were retrospectively included. All the samples were collected as part of the standard care procedures to follow the decrease in the viral load after mAbs administration. Patients’ characteristics were collected through the medical software ORBIS, i.e., age, sex and risk factors for severe forms of COVID-19.

The mAbs procedure was administered once in each patient as a monotherapy (i.e., bamlanivimab) or a combination with two mAbs (i.e., bamlanivimab/etesevimab and casirivimab/imdevimab). Virological monitoring included nasopharyngeal sampling before treatment, and at day 3 (D3) and day 7 (D7) after the administration. For each sample, screening of the E484K mutation was performed with a real-time PCR (TIB MOLBIOL VirSNiP Assay 484K, ref: 53-0789). Then, Sanger sequencing was performed to describe the full gene of the S protein (Table S1). The mutations found in the S gene and the Nextstrain clade were determined for each strain with the Nextclade online tool (https://clades.nextstrain.org/). For each patient, the comparison of strains before mAbs administration and during follow-up was performed.

### Ethics Statement

The study was carried out in accordance with the Declaration of Helsinki. It was a retrospective noninterventional study with no addition to standard care procedures. The reclassification of biological remnants into research material after the completion of the ordered virological tests was approved by the local interventional review board of Pitié-Salpêtrière Hospital. According to the French public health code (CSP article L.1121–1.1), such protocols are exempted from the requirement for individual informed consent.

## 3. Results

In total, thirteen patients were included during the study period, of whom 62% (*n* = 8) were male, with a median (interquartile, IQR) age of 70 (54–79) years old. All the patients had at least one risk factor for progressing to a severe form of COVID-19: four had an active hemopathy under treatment (two had multiple myeloma and were under chemotherapy, one had myelodysplastic syndrome and one had bi-phenotypic, B lymphoid and myeloid acute leukemia for which the patient received a hematopoietic stem cell transplant one year earlier), two had an auto-immune diseases and were under immunosuppressed medications (one had necrotizing myopathy and one had multiple sclerosis) and seven had cardiovascular risk factors (including diabetes, arterial hypertension, dyslipidemia, metabolic syndrome and obesity), of whom one patient also had a heart transplantation in 2016, and one other had renal cancer diagnosed in 2019 and was under chemotherapy at COVID-19 diagnosis. Two patients received a monotherapy of bamlanivimab, three a combination of bamlanivimab/etesevimab and eight a combination of casirivimab/imdevimab (Table 1).

The follow-up was completed for 92% (*n* = 12) of the patients; one patient (P9) did not undergo nasopharyngeal sampling after mAbs administration in our hospital. A total of 49 nasopharyngeal samples were selected, of which eight were not analyzed because of low viral load (*n* = 6) or because there was an insufficient volume to perform the analysis (*n* = 2).

At baseline, the E484K mutation was detected in only one sample issued from patient P6, and Sanger sequencing confirmed the presence of a Variant of Concern (VOC), the Gamma (20J/501Y.V3) variant. In consequence, this patient received a combination of casirivimab/imdevimab, mAbs therapy with bamlanivimab being contraindicated. One patient was infected with a SARS-CoV-2 variant reported in Belgium in March 2021, which was associated with four mutations (Q414K, N450K, D614G and T716I) and an insertion of three amino acids at position 213 (TDR) in the S gene. At last, the remaining strains were identified as VOC Alpha (20I/501Y.V1, *n* = 11). Two of them also harbored polymorphism mutations (P12: K182R and P13: S98F) which remained present during the follow-up (Table 1).

Of the 12 patients with a complete follow-up, one patient had no sample before mAbs administration (P10); however, no mutations were detected in the two samples collected during the follow-up. In total, five mutations (A67V, E406E/G, D427D/Y, Q493R and G1204G/E) appeared in the S gene in samples taken from four patients (P2, P3, P5 and P6), three of whom were receiving bamlanivimab therapy (one as a monotherapy and two in combination) and one a combination of casirivimab/imdevimab. The mutations G1204E and E406G were identified in the mixed population with the wild amino acid and were absent in the following sample. The mutation Q493R was identified in two patients; in patient P2, a mixture of Q493Q/R was detected in the sample collected after the administration of bamlanivimab monotherapy, while in patient P5, four samples harbored the mutation Q493R after the administration of the bamlanivimab/etesevimab combination. The emergence of Q493R was associated with a rise in the SARS-CoV-2 viral load in nasopharyngeal samples (P5: Ct value from 26 on day 19 to 18 on day 68 and P2: Ct from 28 before acquisition of the Q493Q/R to 22 on day 6), as opposed to the mutations G1204E and E406G (Table 1). All the patients recovered from COVID-19, except the two patients with the Q493R-acquired mutations. Clinically, in both of them, the presence of the Q493R mutation was associated with a relapse of COVID-19 with a distress respiratory syndrome. The patient P2 died about two weeks after the emergence of the Q493R, while the patient P5 died about 7 weeks after.

### Description of Patient P5

The patient P5 was diagnosed with acute leukemia (bi-phenotypic, B lymphoid and myeloid) and received a hematopoietic stem cell transplant in May 2020. In the following months, the main complication was a graft-versus-host disease treated with systemic corticosteroids and ciclosporin.

**Figure 1:**
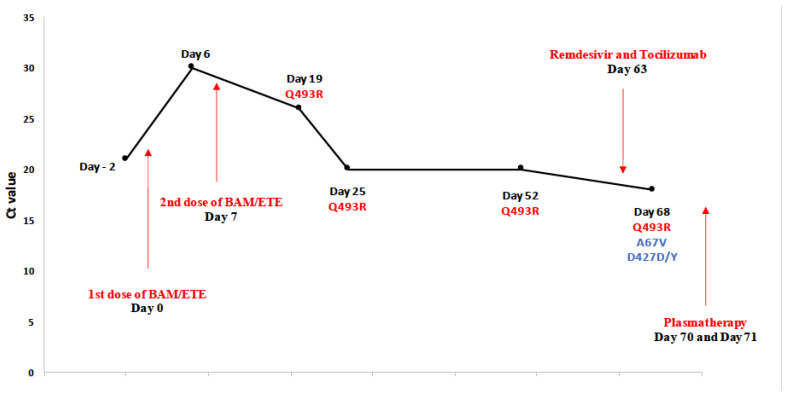
Description of SARS-CoV-2 infection, viral evolution, treatment and outcome in patient P5. This patient was diagnosed with bi-phenotypic acute leukemia and treated with a hematopoietic stem cell transplant in May 2020. A year later, he was admitted to hospital for a pancytopenia associated with hemophagocytic lymphohistiocytosis. He also presented a fever associated with odynophagia and was positive for SARS-CoV-2 infection. After receiving two doses of a combination of bamlanivimab/etesevimab, the spike gene harbored a new mutation (D19), Q493R, which was also present in the following samples (D25, D52 and D68). The acquisition of the Q493R mutation was associated with a new increase in the viral load and a progression to an acute respiratory distress syndrome, leading to hospitalization in an intensive care unit. To face the lack of improvement, remdesivir and tocilizumab were administered on day 63, and then a high titer of convalescent plasma a week later. Following these treatments, two new mutations appeared on day 68 in the spike gene (A67V and D427D/Y). At last, the patient died of COVID-19 a week later.

He was then admitted to hospital for a pancytopenia associated with hemophagocytic lymphohistiocytosis (HLH). Meanwhile, the patient developed a fever, odynophagia and anorexia, and he tested positive for SARS-CoV-2 infection with a Ct value of 21 (D-2). Due to a high risk for progressing to a severe form of COVID-19, he received a combination of bamlanivimab/etesevimab on day 0 and a second dose on day 7, and then was discharged to home. A week later, the fever reappeared, associated with night sweats and diarrhea, and the patient was hospitalized in the medicine department; he was once again diagnosed with an HLH, treated with dexamethasone, and the detection of SARS-CoV-2 in the nasopharyngeal sample remained positive with a Ct value of 26 (D19). At baseline, the VOC Alpha, with its specific pattern of mutations, was identified, and its nucleotide sequence remained the same after the first dose of mAbs. Twelve days after the second injection (D19), the viral load increased again, and this was associated with the appearance of the mutation Q493R, which remained present in all the following samples (D25, D52 and D68). Clinically, the patient progressed to acute respiratory distress syndrome, requiring the hospitalization in an intensive care unit. To face this aggravation, he received remdesivir and tocilizumab on day 63, and then a high titer of convalescent plasma a week later. On day 68, the S gene sequencing revealed the acquisition of two news mutations, A67V and mixed D427D/Y (Figure 1). At last, the patient died as a result of a 2 months of COVID-19.

## 4. Discussion

In the present study, we described the virological evolution of the S gene following the administration of different mAbs therapies to prevent the progression of the disease in patients with mild or moderate forms of COVID-19.

Although we included a low number of patients, we reported the acquisition of the Q493R mutation after bamlanivimab/etesevimab therapy in a patient with bi-phenotypic acute leukemia, associated with a new rise of the SARS-CoV-2 viral load and a fatal outcome. This mutation was previously reported in the literature under the selective pressure of the combination of bamlanivimab/etesevimab in immunosuppressed patients [10,11], especially in the context of B-cell malignancies [12], a similar context to patient P5. The Q493R mutation was also identified in a patient with auto-immune disease, but as part of a mixture. Although we cannot assess the persistence of the mutation over time, a relapse of COVID-19 was observed about two weeks after the emergence of the Q493R mutation and the patient died. In that context, we can postulate that Q493R may have taken over the wild amino acid and may have induced a failure of viral clearance. Our results also corroborate in vitro results observed under selective pressure from bamlanivimab, with the emergence of immune escape mutations, such as E484K and Q493R/K [14,15].

On the other hand, only one of the eight patients under casirivimab/imdevimab therapy developed a mutation in the spike gene, i.e., the E406E/G, which appeared 5 days after mAbs administration; however, its persistence overtime cannot be assessed with a following sample (part of the spike gene sequence was not analyzed). However, no rise of the viral load was observed between the two samples, and the patient recovered from COVID-19, suggesting a limited impact of this mutation on immune escape and resistance, similar to the G1204G/E mutation selected with bamlanivimab/etesevimab.

In late 2021, the emergence of the Omicron variant, which harbored, among others, the Q493R mutation in the spike gene, raised fears of a decline in the efficiency of some monoclonal antibody therapies. Preliminary experiments suggest that most of these treatments are powerless against this new variant excepting, sotrovimab (GSK), approved by the FDA in September 2021, and DXP-604 (BeiGene and Singlomic) [16]. Overall, it would be interesting to develop combinations of antibodies directed against other regions of the spike protein, or at least against other epitopes.

In conclusion, mutations after mAbs administration are a concern and should be monitored, especially when bamlanivimab is administered to immunosuppressed patients, and *a fortiori* in a context of B-cells hemopathy. Furthermore, data are required in the context of the Omicron variant’s emergence on the impact of the new monoclonal antibodies on the spike gene’s evolution.

## Figures and Tables

**Table 1:** Characteristics of the 13 patients treated for a SARS-CoV-2 infection with a specific anti-spike monoclonal antibodies therapy and description of the mutations found in the spike gene at baseline and during follow-up.

		Monoclonal Antibodies					
	Risk Factors of Severe COVID-19 Form	mABS	Time of Sampling	Ct	Nextstrain Clade		Mutation of the Spike Gene
P1	Heart transplantation Corticosteroid-induced diabetes	BAM	D0	17	20B (B1.214.2)		Ins213TDR (ACAGATCGA), Q414K, N450K, D614G, T716I
			D4	20	20B (B1.214.2)		Ins213TDR (ACAGATCGA), Q414K, N450K, D614G, T716I
			D8	29	-		Missing sample
			D9	30	-		Low viral load
						
P2	Necrotizing myopathy	BAM	Pre-administration	33	-		Low viral load
			D0	17	20I (Alpha, V1)		delH69, delV70, delY144, N501Y, A570D, D614G, P681H, T716I, S982A, D1118H
			D4	28	20I (Alpha, V1)		delH69, delV70, delY144, N501Y, A570D, D614G, P681H, T716I, S982A, D1118H
			D6	22	20I (Alpha, V1)		delH69, delV70, delY144, **Q493Q/R**, N501Y, A570D, D614G, P681H, T716I, S982A, D1118H
						
P3	Diabetes type 2 Dyslipidaemia	BAM/ETE	D0	17	20I (Alpha, V1)		delH69, delV70, delY144, N501Y, A570D, D614G, P681H, T716I, S982A, D1118H
			D3	24	20I (Alpha, V1)		delH69, delV70, delY144, N501Y, A570D, D614G, P681H, T716I, S982A, D1118H
			D5	32	20I (Alpha, V1)		delH69, delV70, delY144, N501Y, A570D, D614G, P681H, T716I, S982A, D1118H, **G1204G/E**
			D7	27	20I (Alpha, V1) ^1^		N501Y, A570D, D614G, P681H, T716I, S982A, D1118H
						
P4	Myelodysplastic syndrome Trisomy 8 Corticosteroids	BAM/ETE	D0	21	20I (Alpha, V1)		delH69, delV70, delY144, N501Y, A570D, D614G, P681H, T716I, S982A, D1118H
			D2	19	20I (Alpha, V1)		delH69, delV70, delY144, N501Y, A570D, D614G, P681H, T716I, S982A, D1118H
			D4	24	20I (Alpha, V1)		delH69, delV70, delY144, N501Y, A570D, D614G, P681H, T716I, S982A, D1118H
			D8	33	-		Low viral load
						
P5	Bi-phenotypic acute leukemia GVHD treated with systemic corticosteroids	BAM/ETE	D0	21	20I (Alpha, V1)		delH69, delV70, delY144, N501Y, A570D, D614G, P681H, T716I, S982A, D1118H
			D6	30	20I (Alpha, V1)		delH69, delV70, delY144, N501Y, A570D, D614G, P681H, T716I, S982A, D1118H
			D19	26	20I (Alpha, V1) ^2^		**Q493R**, N501Y, A570D, D614G, P681H, T716I, S982A, D1118H
			D25	20	20I (Alpha, V1)		delH69, delV70, delY144, **Q493R**, N501Y, A570D, D614G, P681H, T716I, S982A, D1118H
			D52	20	20I (Alpha, V1)		delH69, delV70, delY144, **Q493R**, N501Y, A570D, D614G, P681H, T716I, S982A, D1118H
			D68	18	20I (Alpha, V1)		**A67V**, delH69, delV70, delY144, **D427D/Y**, **Q493R**, N501Y, A570D, D614G, P681H, T716I, S982A, D1118H
						
P6	Arterial hypertension, obesity, dyslipidemia, renal cancer under chemotherapy	CAS/IMD	D0	13.5	20J (Gamma, V3)		L18F, T20N, P26S, D138Y, R190S, K417T, E484K, N501Y, D614G, H655Y, T1027I, V1176F
			D2	31	20J (Gamma, V3)		L18F, T20N, P26S, D138Y, R190S, K417T, E484K, N501Y, D614G, H655Y, T1027I, V1176F
			D5	32	20J (Gamma, V3)		L18F, T20N, P26S, D138Y, R190S, **E406E/G**, K417T, E484K, N501Y, D614G, H655Y, T1027I, V1176F
			D7	33	20J (Gamma, V3) ^3^		K417T, E484K, N501Y, D614G, H655Y, T1027I, V1176F
						
P7	Multiple myeloma under chemotherapy	CAS/IMD	D0	16	20I (Alpha, V1)		delH69, delV70, delY144, N501Y, A570D, D614G, P681H, T716I, S982A, D1118H
			D4	29	20I (Alpha, V1)		delH69, delV70, delY144, N501Y, A570D, D614G, P681H, T716I, S982A, D1118H
			D6	30	20I (Alpha, V1)		delH69, delV70, delY144, N501Y, A570D, D614G, P681H, T716I, S982A, D1118H
			D8	21	20I (Alpha, V1)		delH69, delV70, delY144, N501Y, A570D, D614G, P681H, T716I, S982A, D1118H
			D13	36	-		Low viral load
						
P8	Multiple myeloma under chemotherapy	CAS/IMD	D0	21	20I (Alpha, V1)		delH69, delV70, delY144, N501Y, A570D, D614G, P681H, T716I, S982A, D1118H
			D0Bis	18	20I (Alpha, V1)		delH69, delV70, delY144, N501Y, A570D, D614G, P681H, T716I, S982A, D1118H
			D1	16.5	20I (Alpha, V1)		delH69, delV70, delY144, N501Y, A570D, D614G, P681H, T716I, S982A, D1118H
			D9	33	-		Low viral load
						
P9	Age > 80 years, obesity	CAS/IMD	D0	17	20I (Alpha, V1)		delH69, delV70, delY144, N501Y, A570D, D614G, P681H, T716I, S982A, D1118H
						
P10	Diabetes type 2 Arterial hypertension Metabolic syndrome Obesity (BMC = 35.8)	CAS/IMD	D0	16	-		Missing sample
			D4	25	20I (Alpha, V1) ^4^		N501Y, A570D, D614G, P681H, T716I, S982A, D1118H
			D6	30	20I (Alpha, V1)		**T20I**, delH69, delV70, delY144, N501Y, A570D, D614G, P681H, T716I, S982A, D1118H
						
P11	Age > 80 years Arterial hypertension Chronic obstructive bronchopneumopathy	CAS/IMD	D0	25	20I (Alpha, V1)		delH69, delV70, delY144, N501Y, A570D, D614G, P681H, T716I, S982A, D1118H
			D3	31	20I (Alpha, V1)		delH69, delV70, delY144, N501Y, A570D, D614G, P681H, T716I, S982A, D1118H
			D5	30	20I (Alpha, V1)		delH69, delV70, delY144, N501Y, A570D, D614G, P681H, T716I, S982A, D1118H
			D7		20I (Alpha, V1) ^5^		delH69, delV70, delY144, N501Y, A570D, D614G
						
P12	Diabetes type 2 Vascularitis Renal transplantation	CAS/IMD	D0	14	20I (Alpha, V1)		delH69, delV70, delY144, **K182R**, N501Y, A570D, D614G, P681H, T716I, S982A, D1118H
			D4	25	20I (Alpha, V1)		delH69, delV70, delY144, **K182R**, N501Y, A570D, D614G, P681H, T716I, S982A, D1118H
			D8	22	20I (Alpha, V1)		delH69, delV70, delY144, **K182R**, N501Y, A570D, D614G, P681H, T716I, S982A, D1118H
						
P13	Multiple sclerosis under immunosuppressor treatment (Ocrelizumab)	CAS/IMD	pre-administration	17	20I (Alpha, V1)		delH69, delV70, **S98F**, delY144, N501Y, A570D, D614G, P681H, T716I, S982A, D1118H
			D0	20	20I (Alpha, V1)		delH69, delV70, **S98F**, delY144, N501Y, A570D, D614G, P681H, T716I, S982A, D1118H
			D7	33		-	Low viral load

D: day from the administration of monoclonal therapies; GVHD: graft-versus-host disease; BAM: bamlanivimab; ETE: etesevimab; CAS: casirivimab; IMD: imdevimab; Ct: cycle threshold; del: deletion; Ins: insertion; **bold**: polymorphism mutations; **red**: resistance mutations already described in the literature; **blue**: mutations of which resistance impact is unknown. ^1^ Part of the spike gene sequence not analyzed: AA1 to AA176. ^2^ Part of the spike gene sequence not analyzed: AA1 to AA396. ^3^ Part of the spike gene sequence not analyzed: AA1 to AA410. ^4^ Part of the spike gene sequence not analyzed: AA1 to AA175. ^5^ Part of the spike gene sequence not analyzed: AA655 to AA1274. GenBank accession numbers: P1_D0: OL405044; P1_D4: OL405045; P2_D0: OL405046; P2_D4: OL405047; P2_D6: OL405048; P3_D0: OL405049; P3_D3: OL405050; P3_D5: OL405051; P3_D7: OL405052; P4_D0: OL405053; P4_D2: OL405054; P4_D4: OL405055; P5_D0: OL405056; P5D6: OL405057; P5_D19: OL405058; P5_25: OL405059; P5_52: OL405060; P5_68: OL405061; P6_D0: OL405062; P6_D2: OL405063; P6_D5: OL405064; P6_D7: OL405065; P7_D0: OL405066; P7_D4: OL405067; P7_D6: OL405068; P7_D8: OL405069; P8_D0: OL405070; P8_D0bis: OL405071; P8_D1: OL405072; P9_D0: OL405073; P10_D4: OL405074; P10_D6: OL405075; P11_D0: OL405076; P11_D3: OL405077; P11_D5: OL405078; P11_D7: OL405079; P12_D0: OL405080; P12_D4: OL405081; P12_D8: OL405082; P13_D-1: OL405083; P13_D0: OL405084.

## Data Availability

All the Spike gene sequences are publicly available on NCBI GenBank and registered with the following number: P1_D0: OL405044; P1_D4: OL405045; P2_D0: OL405046; P2_D4: OL405047; P2_D6: OL405048; P3_D0: OL405049; P3_D3: OL405050; P3_D5: OL405051; P3_D7: OL405052; P4_D0: OL405053; P4_D2: OL405054; P4_D4: OL405055; P5_D0: OL405056; P5D6: OL405057; P5_D19: OL405058; P5_25: OL405059; P5_52: OL405060; P5_68: OL405061; P6_D0: OL405062; P6_D2: OL405063; P6_D5: OL405064; P6_D7: OL405065; P7_D0: OL405066; P7_D4: OL405067; P7_D6: OL405068; P7_D8: OL405069; P8_D0: OL405070; P8_D0bis: OL405071; P8_D1: OL405072; P9_D0: OL405073; P10_D4: OL405074; P10_D6: OL405075; P11_D0: OL405076; P11_D3: OL405077; P11_D5: OL405078; P11_D7: OL405079; P12_D0: OL405080; P12_D4: OL405081; P12_D8: OL405082; P13_D-1: OL405083; P13_D0: OL405084.

## Supplementary Materials

The following supporting information can be downloaded at: https://www.mdpi.com/article/10.3390/v14020226/s1, Table S1: Experimental conditions for the amplification and sequencing of the full sequence of the Spike gene.
