# Supplementary material for: Spike Gene Evolution and Immune Escape Mutations in Patients with Mild or Moderate Forms of COVID-19 and Treated with Monoclonal Antibodies Therapies
Source: Viruses. 2022 Jan 24;14(2):226. doi: 10.3390/v14020226 (PMC8877338; doi:10.3390/v14020226)
Supplement: Supplementary file 1 [file viruses-14-00226-s001.zip › viruses-1564954-supplementary.pdf]

Table S1 : Experimental conditions for the amplification and sequencing of the full sequence of the Spike gene

Primers sequences

**Amplicon A**	**Primers**	**Sequence 5' – 3'**	**PCR products (size, bp)**
1st PCR	Forward	AGGGGTACTGCTGTTATGTCT	2192
	Reverse	CCCGCCGAGGAGAATTAGTC	
Nested PCR A1	Forward	AACAACAGAGTTGTTATTTCTAGTGA	Around 800
	Reverse	CCTGAGGGAGATCACGCAC	
Nested PCR A2	Forward	TCTCTCAGCCTTTTCTTATGGACC	Around 800
	Reverse	TTCCAAGCTATAACGCAGCCT	
Nested PCR A3	Forward	TCTGCTTTACTAATGTCTATGCAGA	Around 800
	Reverse	GTTGTTGACATGTTCAGCCCC	
**Amplicon B**	**Primers**	**Sequence 5' – 3'**	**PCR products (size, bp)**
1st PCR	Forward	TGCACAGAAGTCCCTGTTGC	2112
	Reverse	CGAAAGGGAGTGAGGCTTGT	
Nested PCR B1	Forward	AACTTACTCCTACTTGGCGTGT	Around 800
	Reverse	CATCTGTGAGCAAAGGTGGC	
Nested PCR B2	Forward	CACTTGCAGATGCTGGCTTC	Around 800
	Reverse	CTTCACGAGGAAAGTGTGCT	
Nested PCR B3	Forward	CCCTCAGTCAGCACCTCATG	Around 800
	Reverse	GCATCCTTGATTTCACCTTGC	

Amplification program

	**Thermal profile**	**Cycle**	**Time**	**Temperature**
**1st PCR Amplicon A and B**	Reverse transcription	1	45 min	45°C
	Initial denaturation	1	2 min	95°C
	Denaturation	40	30 s	95°C
	Annealing		30 s	55°C
	Elongation		2 min	68°C
	Final elongation	1	7 min	68°C
	Cooling	1	indefinitely	10°C
**Nested PCR Amplicon A1, A2, A3, B1, B2 and B3**	**Thermal profile**	**Cycle**	**Time**	**Temperature**
	Initial denaturation	1	5 min	94°C
	Denaturation	45	30 s	94°C
	Annealing		30 s	55°C
	Elongation		1 min	72°C
	Final elongation	1	7 min	72°C
	Cooling	1	indefinitely	10°C
